# Genomics and breeding innovations for enhancing genetic gain for climate resilience and nutrition traits

**DOI:** 10.1007/s00122-021-03847-6

**Published:** 2021-05-20

**Authors:** Pallavi Sinha, Vikas K. Singh, Abhishek Bohra, Arvind Kumar, Jochen C. Reif, Rajeev K. Varshney

**Affiliations:** 1 International Crops Research Institute for the Semi-Arid Tropics (ICRISAT), Hyderabad, India; 2 International Rice Research Institute (IRRI), IRRI South Asia Hub, ICRISAT, Hyderabad, India; 3 ICAR- Indian Institute of Pulses Research (IIPR), Kanpur, India; 4 Leibniz Institute of Plant Genetics and Crop Plant Research (IPK), Gatersleben, Germany; 5 State Agricultural Biotechnology Centre, Centre for Crop and Food Innovation, Food Futures Institute, Murdoch University, Murdoch, WA Australia

## Abstract

**Key message:**

Integrating genomics technologies and breeding methods to tweak core parameters of the breeder’s equation could accelerate delivery of climate-resilient and nutrient rich crops for future food security.

**Abstract:**

Accelerating genetic gain in crop improvement programs with respect to climate resilience and nutrition traits, and the realization of the improved gain in farmers’ fields require integration of several approaches. This article focuses on innovative approaches to address core components of the breeder’s equation. A prerequisite to enhancing genetic variance (*σ*^*2*^*g*) is the identification or creation of favorable alleles/haplotypes and their deployment for improving key traits. Novel alleles for new and existing target traits need to be accessed and added to the breeding population while maintaining genetic diversity. Selection intensity (*i*) in the breeding program can be improved by testing a larger population size, enabled by the statistical designs with minimal replications and high-throughput phenotyping. Selection priorities and criteria to select appropriate portion of the population too assume an important role. The most important component of breeder′s equation is heritability (*h*^*2*^). Heritability estimates depend on several factors including the size and the type of population and the statistical methods. The present article starts with a brief discussion on the potential ways to enhance *σ*^*2*^*g* in the population. We highlight statistical methods and experimental designs that could improve trait heritability estimation. We also offer a perspective on reducing the breeding cycle time (*t*), which could be achieved through the selection of appropriate parents, optimizing the breeding scheme, rapid fixation of target alleles, and combining speed breeding with breeding programs to optimize trials for release. Finally, we summarize knowledge from multiple disciplines for enhancing genetic gains for climate resilience and nutritional traits.

## Introduction

Extreme weather and precipitation events, decreasing soil fertility and plant productivity, and rising disease and pest pressure and crop failures resulting from climate change threaten global agricultural production (Dhankher and Foyer 2018; Acevedo et al. 2020). The 0.74 °C rise in the average global temperature during the last 100 years is likely to go up by 2.6–4.8 °C by the end of this century (Leisner 2020). Climate change-led decline in agricultural production jeopardizes the food security of the global population. Globally, 794.6 million people suffer from undernourishment (FAO 2015) and nutrition-related problems account for 45% of deaths in children under 5 years of age (Salam et al. 2015). The situation is alarming in developing regions of the world that harbor 779.9 million-undernourished people. Reduced agricultural production leading to food shortage and food inflation is likely to profoundly impact the future food supply to the people inhabiting the low-income regions (Islam and Wong 2017). The current genetic gain rates achieved so far in major agricultural crops remain insufficient to meet the required food demands (Cooper et al. 2020). Improving the rate of genetic gain in crop breeding programs could accelerate the delivery of crop cultivars with climate resilience and higher nutrient density, which will be crucial for sustainable food production and food security of the growing population.

Genetic gain is the incremental improvement in the trait achieved in a breeding program, and the unit of time is per generation. It is an important dimension contributing to improvements in grain yield productivity and food security under changing climatic conditions (Fischer et al. 2014). Based on the breeder’s equation, the genetic gain is indirectly proportional to breeding cycle time (*t*) and depends on the genetic variation (*σ*^*2*^_*g*_), the intensity of selection (*i*), and heritability of the trait (*h*^*2*^). Enhancing genetic gain in breeding programs and its realization in farmers’ fields calls for an integration of multiple aspects including germplasm resources, genomics, breeding, and agronomic practices in concern with improved seed delivery systems (Varshney et al. 2018).

Innovations in high-throughput genotyping, phenotyping and informatics tools present an enormous opportunity to revisit the breeder’s equations in terms of genetic gain over time. Growing phenotyping capacities such as disease sick plots, artificial screening in laboratories or greenhouse can help to screen a large number of plants for the trait of interest in a short period of time. Molecular markers can serve as proxies for target phenotypes allowing selection to be performed on young plants and/or in early generations. Gene-based/linked markers allow selection in “off-target” years and at any place and in any given situation. The availability of cost-effective and high-throughput genotyping platforms can allow the assaying of thousands of plants in a relatively short time. Breeding cycle time (*t*) can be reduced by harvesting more generations per year, instead of the usual one or two cycles per year, and making selection on a single plant basis, using visual selection or molecular markers.

Integration of genomics tools for improving the efficiency of plant breeding has been referred to as genomics-assisted breeding (GAB, Varshney et al. 2005, 2021). The GAB approaches including marker-assisted backcrossing (MABC), marker-assisted recurrent selection (MARS), and genomic selection (GS) have been in use in breeding programs (Varshney et al. 2021). MABC is the standard technique to introgress a few loci or major QTLs for improving elite varieties. In the MARS approach, a number of loci (genomic regions) are identified from an elite × elite cross and then superior alleles are assembled into one genetic background. GS enables the evaluation of the genetic worth of an individual based on genomic estimated breeding values (GEBVs) that are calculated by using genome-wide marker profiling data and intensive phenotypic information (Crossa et al. 2017). In the context of enhancing the effectiveness of GAB approaches by using the current innovations in genomics, breeding and other allied disciplines, we propose to address each component of the breeder’s equation for accelerating genetic gain to climate resilience and nutrition traits.

We begin by highlighting the potential ways to enhance genetic variance (*σ*^*2*^_*g*_) through the identification and creation of favorable alleles and their deployment in the breeding population. This is followed by a discussion on selection intensity (*i*), and in this section, we have covered various aspects including population size, proportion of selected individuals, selection criteria (single/multi-trait), selection methods (phenotypic, marker-based, and combined selection), selection priorities (must have and value-added traits), and selection targets (hotspots/glasshouse/target population of environments, TPEs). In the third section, we have discussed how to estimate and increase the heritability (*h*^*2*^) of the component traits. We have considered several methods of statistical analyses, and experimental designs to estimate the heritability. And in the last, we proposed reducing the breeding cycle time (*t*) through the selection of appropriate parents, optimizing the breeding scheme, rapid fixation of target alleles, and integrating speed breeding approach into the main breeding programs.

### Introducing genetic variation

**Fig. 1:**
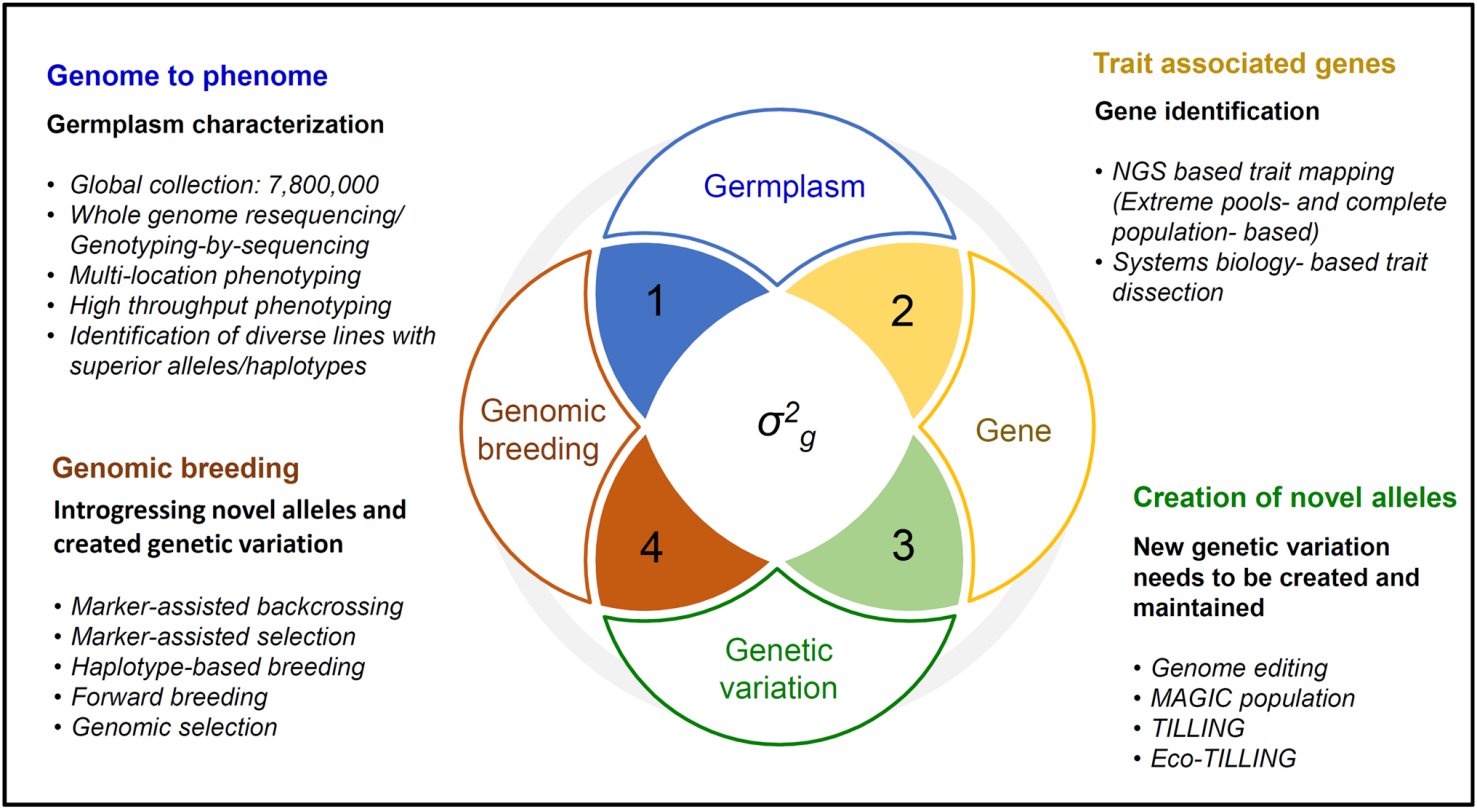
Enhancing genetic variation through identifying/ creating and utilizing favorable alleles/haplotypes. Genetic variance (*σ*^*2*^_*g*_) is estimated with genetic parameters including phenotypic variance composed by genotypic and environmental variance (not inheritable). Genetic variance allows an understanding of the genetic structure involved in the progenies, determined by additive and non-additive effects. (1) Genome to phenome deals with the connection and causation between the genetic makeup of an accession and the observed physical or physiological traits or characteristics (phenome). This can be achieved by characterizing germplasm collections at the phenotypic and genotypic level. (2) Trait associated genes can be identified through NGS based trait mapping (extreme pools- based and complete population-based) and/or through systems biology approaches. (3) Genetic variations can be assayed using germplasm characterization or can be created by through genome editing, multiparent advanced generation intercross (MAGIC), targeting induced local lesions in genomes (TILLING) and Eco-TILLING populations. (4) Genomic breeding to combine superior/ novel alleles/ haplotypes in elite backgrounds. The identified genetic variations can be introduced in a crop improvement programs through genomics-assisted breeding (GAB) approaches including marker- assisted backcrossing (MABC), marker- assisted recurrent selection (MARS), haplotype-based breeding (HBB), forward breeding (FB) and genomic selection (GS).

For enhancing and maintaining *σ*^*2*^_*g*_ in the breeding population, modern breeding and genomics technologies and advanced phenotyping platforms can be utilized. The germplasm collections and specialized populations would be a useful source to identify superior alleles, haplotypes, or genes for climate resilience and nutrition traits. These novel genetic variations can be introduced and maintained in breeding programs by GAB approaches (Fig. 1).

#### Identification of superior alleles and haplotypes

Genebanks provide a long-term mission of preserving plant genetic resources as an agricultural legacy for future crop improvement. Worldwide germplasm collections of approximately 7.4 million accessions are stored and preserved in more than 1750 gene banks (FAOSTAT 2010). In this context, McCouch et al. (2013, 2020) suggested mining the crop diversity for target traits present in these gene banks. Since the germplasm collections in several crops are very large, it is quite challenging to perform precise and accurate phenotyping of these sets. There have been several efforts to characterize large germplasm sets at the sequencing level though. In this regard, different types of customized germplasm sets such as core collection, mini-core collection, reference set, FIGS (focused identification of germplasm strategy) have been developed (Upadhyaya and Ortiz 2001; Stenberg and Ortiz 2020). Nevertheless, next-generation sequencing (Varshney et al. 2019a) and genotyping technologies (Rasheed et al. 2017) combined with large-scale phenotyping (Mir et al. 2012; Jin et al. 2020) have been utilized in a number of plant species for the identification of marker-trait associations (MTAs). Examples include rice (Huang et al. 2010), wheat (Neumann et al. 2011), foxtail millet (Jia et al. 2013), pigeonpea (Varshney et al. 2017a), pearl millet (Varshney et al. 2017b), chickpea (Varshney et al. 2013, 2019b), rapeseed (Wu et al. 2018; Lu et al. 2019), cotton (Du et al. 2018; Ma et al. 2018), foxtail millet (Jia et al. 2013), and grapevine (Liang et al. 2019). These studies shed new insights on the genetic architecture of agriculturally important traits and reveal valuable and sometimes novel alleles for morphological, agronomic, developmental, and quality-related traits. Furthermore, whole-genome sequencing efforts have also been employed to sequence 3180 rice accessions (Wang et al. 2018), and 3366 accessions of chickpea (Varshney et al. unpublished) that provide genome-wide information on these germplasms for use in the breeding programs. However, sequencing of large germplasm collection becomes quite expensive in species with large genome size like wheat and barley. Therefore, cost-efficient methods based on reduced representation sequencing protocols have been employed to characterize 44,624 wheat breeding lines (Juliana et al. 2019) and 22,626 wild and domesticated barley accessions (Milner et al. 2019). These are initial examples showing the power of genomics and informatics technologies to characterize large populations or even entire germplasm collections (Langridge and Waugh 2019). Not only sequencing-based germplasm characterization, in some crops like wheat, ICAR- National Bureau of Plant Genetic Resources (NBPGR), India, has shown a possibility of phenotyping of 22,416 accessions of wheat for 23 qualitative and 12 quantitative traits (Phogat et al. 2021). Recent advances in pangenomics including the development of super-pan genomes are expected to accelerate trait mapping and identify novel alleles from the germplasm collection (Khan et al. 2020).

Multiparent populations including nested association mapping (NAM) and multi-parent advanced generation inter-cross (MAGIC) are new experimental designs that combine allele richness, high mapping resolution, and high statistical power of both linkage analysis and association mapping (Huang et al. 2015; Scott et al. 2020). Both types of populations have been successfully developed and used to identify QTLs for a number of traits in diverse crop species (see Bohra et al. 2020a). Several successful examples of utilizing NAM for dissecting the traits of interest have been reported in maize (Yu et al. 2008), durum wheat (Kidane et al. 2019), and barley (Sharma et al. 2018). Also, recent literature highlights the growing relevance of MAGIC populations for trait mapping in crops like wheat (Stadlmeier et al. 2018), rice (Bandillo et al. 2013), cotton (Thyssen et al. 2019), cowpea (Huynh et al. 2018), tomato (Gonda et al. 2019), and maize (Septiani et al. 2019). Nevertheless, the power of bi-parental mapping populations for trait/gene discovery has also been enhanced several fold owing to the high-density genotyping of entire populations (Bohra et al. 2020a).

Characterization of germplasm and specialized genetic populations can help to identify or create novel and superior alleles or haplotypes as well as donors for specific traits of interest for use in crop improvement. For instance, genome-wide association analyses on germplasm sets/populations have elucidated the genetic architecture, marker-trait association, and haplotypes for climate resilience traits, *i.e.,* drought, heat, and salinity stresses in chickpea (Li et al. 2018; Varshney et al. 2019a), pearl millet (Varshney et al. 2017b), pigeonpea (Sinha et al. 2020a), soybean (Patil et al. 2016; Do et al. 2019), wheat (Neumann et al. 2011), etc. Besides, germplasm sequencing provides the genome-wide information on domestication phenomenon and extent of genetic load, i.e., deleterious mutations in the elite germplasm (Ramu et al. 2017; Varshney et al. unpublished). The accurate information on genomic loci underlying domestication traits opens new avenues for de novo domestication of wild species for designing ideal crops for sustainable agriculture (Fernie and Yan 2019).

#### NGS-based rapid gene identification

Advances in genomics have facilitated a variety of NGS-based rapid trait mapping approaches like QTL-seq, MutMap, Indel-Seq, or BSR-Seq (Singh et al. 2016; Pandey et al. 2017). NGS technologies have enabled modification and improvement of traditionally tricky, time-consuming bulked segregant analysis (Michelmore et al. 1991) into rapid and whole-genome sequencing-based high-resolution trait mapping strategy (Schlötterer et al. 2014; Mascher et al. 2014). For instance, Takagi et al. (2015) identified *hitomebore salt-tolerant 1* (*hst1*) locus in rice following the MutMap approach, and the study demonstrated its immediate utility in rice genetic improvement through breeding salt-tolerant rice Kaijin carrying the recessive *hst1* allele in just two years.

Sequencing-based trait mapping combines both classical genetics and NGS platforms to map the breeding traits such as climate resilience and nutrition at a higher resolution. Based on the sequencing strategy, sequencing-based trait mapping can be broadly grouped into two classes: a) trait mapping through pooled sequencing of populations and b) trait mapping through sequencing of the entire population. Examples of NGS-based trait mapping have been reported in many crop species (see Varshney et al. 2018). With the availability of draft genomes for the majority of crops, including so-called orphan crops such as chickpea (Varshney et al. 2013), pigeonpea (Varshney et al. 2012), groundnut (Bertioli et al. 2016, 2019; Chen et al. 2019; Zhuang et al. 2019), and reduced costs on sequencing coupled with availability of data analysis pipelines, we anticipate accelerated growth in trait mapping including climate resilience and nutrition traits (see Varshney et al. 2019b).

Efforts to understand the regulatory mechanisms of complex plant traits have gained impetus with the recent advances in systems biology (see Sehgal et al. 2018). Systems biology is a holistic approach that improves understanding of the biological systems by integrating multiple -omics approaches (genomics, transcriptomics, epigenomics, proteomics, metabolomics) with modeling, synthetic biology, and high-performance computational analysis (Lavarenne et al. 2018; Pazhamala et al. 2021). In brief, systems biology is the study of a trait, viewed as an integrated and interacting network of genes, proteins, and biochemical reactions. The goal of systems biology is to discover new emergent properties to understand better the entirety of processes that happen in a biological system. To enhance systems biology and better inform breeding decisions, gene expression atlas (Nobuta 2007; Pazhamala et al. 2017; Kudapa et al. 2018; Shinozaki et al. 2018; Hoopes et al. 2019; Sinha et al. 2020b), and maps based on epigenome (Li et al. 2019; Junaid et al. 2018; Peng et al. 2019; Sinha et al. 2020c), proteome (Barua et al. 2019; Duncan et al. 2017; Jiang et al. 2019), and metabolome (Okazaki and Saito 2016; Chen et al. 2018) have been developed in many crops in addition to the existing saturated genome maps. The systems biology approach should be specifically targeted to understand the molecular mechanism of complex traits related to climate resilience such as drought tolerance (Miao et al. 2017) as improving these traits will require deep knowledge at the systems level (Pazhamala et al. 2021).

#### Introgressing novel alleles and creating genetic variation

Once useful genetic variation is identified or created for breeding traits, GAB approaches can be used to deploy them in crop improvement (Varshney et al. 2021). The forward breeding (FB) approach is the best solution when early generation selection has to be done for ‘must-have traits.’ In this approach, DNA markers associated with the trait of interest can be assayed in the early segregating populations. Selected plants with the gene(s) of interest can be advanced to the next generations while the remaining plants can be discarded. The FB approach gained momentum with the establishment of a high-throughput genotyping (HTPG) project that offers to genotype a line (with the DNA extraction cost included) at affordable cost, *i.e.,* US$ 1.5 (Bohar et al. 2020a, Bohra et al. 2020b).

If a breeder wishes to combine different loci in an elite genetic background, then the following two approaches can be implemented in the program based on the number of loci. MABC can be useful to introgress a few loci (> 10 loci) for improving elite varieties. This approach has been used extensively to develop a large number of improved varieties for commercial release in the public and private sectors. Compared to MABC, MARS is better equipped to address complex traits and has been proven useful to introgress as many as 40 loci through intercrossing elite × elite parents (Bernardo and Charcosset 2006). This approach can be used to develop superior lines with an optimum combination of superior alleles through repeated inter-crossing.

As mentioned above, large-scale sequencing and extensive phenotyping of germplasm collection can provide various haplotypes including ‘superior haplotypes’ for the target traits (Bevan et al. 2017). ‘Superior haplotypes' explain the phenotypic performance of the group of individuals (specific haplotype group) that remains significantly superior to the other haplotype group (Sinha et al. 2020a). ‘Superior haplotypes’ can be introduced in a crop improvement program through haplotype-assisted forward breeding (in the case superior haplotype exists in elite pool) or haplotype-assisted backcross breeding (in the case superior haplotype doesn’t exist in the elite pool but present in the landraces).

#### Genomic selection

The next-generation molecular marker systems and sequencing technologies paved the way for GS in animals and plants (Meuwissen 2009). GS exploits genome-wide genetic marker data to predict the phenotype and offers many advantages, including a drastic cost reduction in the repeated phenotyping (Meuwissen et al. 2001). GS has a high accuracy of prediction in elite genetic materials through genomic estimated breeding values (GEBVs) even in the initial generations and enables shortening of breeding cycles (Crossa et al. 2017).

The GS models are very much useful in predicting hybrid performance in crops. For example, for predicting hybrid performance in oilseed rape, Werner et al. (2018) computed general combining ability (GCA) and specific combining ability (SCA) based on RR-BLUP and Bayesian models. Vélez-Torres et al. (2018) advocated for GS as a more effective and efficient approach to predicting maize lines' GCA than the conventional phenotype-based methods. For dry matter yield in maize, Riedelsheimer et al. (2012) also found that compared to traditional phenotypic selection for GCA, SNP-based GS of the parental inbred lines in the whole population reached a relative efficiency of 0.83 in less time and without multi-environment field trials of the testcrosses. Crossa et al. (2013) detailed that GS could be applied in predicting the genetic worth of breeding lines for potential release as cultivars or predicting the breeding values of candidates in rapid-cycle populations through accurate predictions of additive effects in the early generations.

Of the various factors that influence GS application in plants, one is to incorporate genotype (genomic) × environment interaction (G × E) for specific environments or G × E interactions for multi-traits into statistical models in predicting unobserved individuals. Understanding the complexity of traits requires a theoretical framework that accounts for often cryptic interactions. Usually, additive genetic effects can be predicted by Bayesian inference (Meuwissen et al. 2001) and to develop the genomic relationship linear kernel matrix (G) to fit the GBLUP (VanRaden 2008). The GBLUP is flexible enough to be extended to more complex situations like incorporating G × E interactions.

##### Genomic prediction incorporating G × E

The prediction accuracies of the GS models can be improved through the inclusion of G × E effects, and in recent years, several GS models were developed and deployed to account for the G × E effects. For instance, Jarquín et al. (2014) proposed an extension of the GBLUP, a random-effects model that includes both main effects of markers and environmental covariates (ECs) and their interactions with the help of covariance structures that are functions of marker genotypes and ECs. The marker × environment interaction model by Lopez-Cruz et al. (2015) partitions the marker effects and genomic values into components that are stable across environments (main effects) and others that are environment-specific (interactions). This interaction model helps in selecting for stability and for adaptation to targeted environments. This model is compatible with standard GS software and with commonly used GS parameters and approaches, including shrinkage methods (e.g., GBLUP) and variable selection methods (that is otherwise difficult to implement directly with the normal model) (Crossa et al. 2016). Further, Cuevas et al. (2016) modified the earlier proposed model of Lopez-Cruz et al. (2015) and applied both the standard linear kernel (GBLUP) and a nonlinear Gaussian kernel similar to that used in the RKHS (reproducing kernel Hilbert spaces). Besides, the large number of individuals and G × E sometimes make a GS model difficult and computationally very intensive. The BGGE (Bayesian Genomic Genotype × Environment interaction) software of Granato et al. (2018) can manage this situation, but it comes with a high computational cost. Overcoming this for large data, Cuevas et al. (2020) applied an approximate kernel method following a Bayesian approach in BGLR, a genomic-enabled prediction R (Pérez-Rodríguez and de los Campos 2014). This model reduced the computing time and showed a competitive prediction performance of the approximated methods.

##### Selection indices in genomic selection

Genomic selection index (GSI), a linear combination of GEBVs, combines phenotypic and GEBV information to predict the net genetic merit of the unobserved individuals (Ceron-Rojas et al. 2015). The conditions for constructing a valid linear GSI are that all marker effects should be estimated simultaneously in the training population. The estimated effects should be used in subsequent selection cycles to obtain GEBV that predicts individual breeding values in the testing population for which there is only marker information available. Lande and Thompson (1990) have proposed incorporation of marker information into selection index theory through combining marker information with phenotypic information. Later, Dekkers (2007) suggested that GEBVs can be incorporated into selection index model to predict response and rate of inbreeding. In the context of GS, the GEBV-only index and GEBV-assisted index were subsequently proposed by Togashi et al. (2011), in which the former combined GEBVs for two traits and later combines GEBV of one trait with traditional BLUP selection for another trait remain largely hypothetical owing to the lack of support from empirical or simulation data. The study by Ceron-Rojas et al. (2015) implemented GSI to predict the GS response and genetic gain per selection cycle for unobserved traits after the first selection cycle. Results from Ceron-Rojas et al. (2015) based on both simulated and real data contributed to establish the efficiency of GSI over phenotypic selection indices. The efficiency of GSI models has been further supported from evidence in several crops including wheat, maize, rice, sorghum (see Habyarimana et al. 2020).

#### Genome editing

Although transgenic technology has been used in the past for targeting climate resilience traits, only limited success has been achieved (Varshney et al. 2011). Genome/gene editing (GE) has emerged as a powerful approach for improving plant performance and the development of various abiotic and biotic stress tolerance lines. This field has developed enormously thanks to the new *Cas* and GE methods supported with appropriate bioinformatics pipelines. To date, a large number of genes with significant phenotypic effects have been cloned and functionally characterized in many crops. Genes with defined sizable phenotypic effects can be utilized through the GE approach, referred to as the promotion of alleles by genome editing (PAGE) (Jenko et al. 2015). GE approach can also be used to purge deleterious alleles and the approach is referred to as the removal of alleles by genome editing (RAGE). Crop-specific information on functional genes has been utilized to develop GE crops and > 60 successful examples have been reported to date for traits like herbicide tolerance, disease resistance, drought tolerance, enhanced oil quality, improved cell wall expansion, etc. (Zhang et al. 2018).

### Enhancing selection intensity

**Fig. 2:**
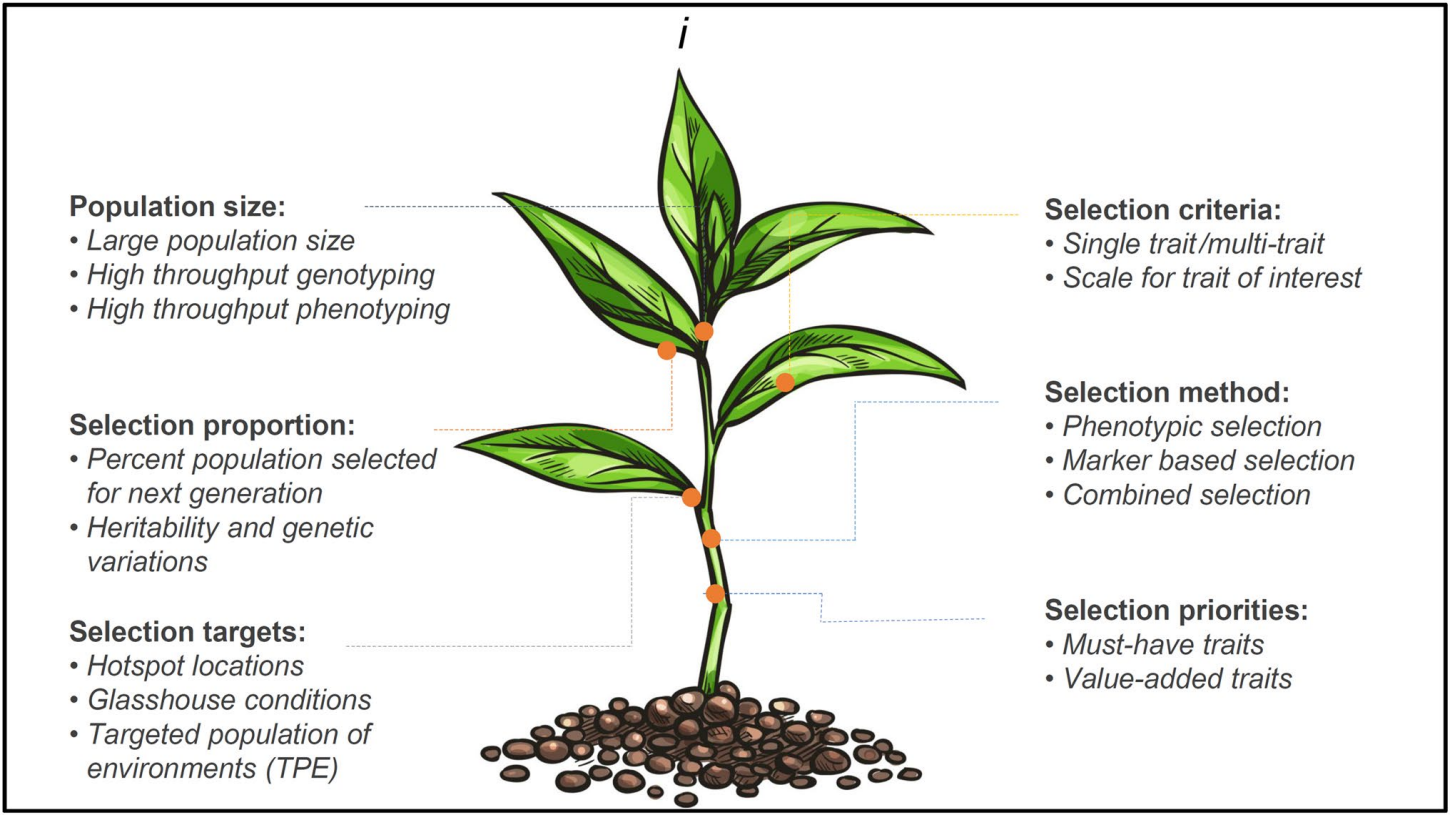
Enhancing selection intensity *(i)* through tweaking various parameters. For instance, the population size should be large enough to carry desirable recombinants for the trait of interest. As a matter of fact, the molecular markers can only be useful to identify the recombinants but cannot create recombinants into the populations. The effects of selection ratio (*k*) and phenotypic variation (standard deviation, *σ*) on selection differential also is an important factor. Selection targets can also be an important factor for the selection of traits including selection on hotspots, or selection in glasshouse conditions, and most importantly selection of traits in target population of environments (TPEs). High-throughput phenotyping and statistical designs can be useful to enhance selection intensity. Selection criteria is another important factor in which selection is based upon single or multiple traits and at the same time, the scale defined to characterize the traits is another important measure. Method of selection is a critical factor to increase the overall selection intensity which includes selection through trait-associated markers or a combination of phenotypic and genotypic selections. Another important basis for selection is the priorities of traits that can be characterized as must-have and value-added traits. In summary the selection intensity in breeding program depends upon the number of traits to be selected at the different stages, which requires rational budgeting of resources to handle populations with large sizes

Accumulating favorable alleles in a population or restricting the transmission of the unfavorable alleles to the next generation determines the rate of genetic improvement (Fig. 2). This is achieved by managing the proportion of individuals selected as parents for the next cycle, reflected in the form of selection differential (*S*), *i.e.,* the difference of means of selected individuals over the mean of the non-selected population. Selection intensity (*i*) defined as standardized selection differential can be increased through i) enhancing experimental scales and populations size, ii) reducing the proportion selected for the next generation, and iii) improving accuracy in selection targets, selection methods, and with better-defined selection priorities (must have and value-added traits) (Fig. 2). Optimized selection intensity is, however, warranted to strike a balance between selection differential and inbreeding depression since higher selection intensity may improve gain in the short term but faces a challenge to sustain in the long-term owing to a reduction in genetic variation (Rumball and Rae 1968).

#### Effective population size

A crucial factor that forms the basis of selection intensity is the population size. Higher population size is related to higher selection response given the fact that the chances of losing favorable alleles on higher selection intensity are greatly reduced in comparison with smaller populations. Effective population size is depending upon the rate of genetic drift, *i.e.,* the shift of allele frequencies that occurs due to the sampling of alleles that are contributed to progeny. Genetic drift causes non-independence of alleles at different loci (linkage disequilibrium, LD). This LD allows markers to predict the phenotype. The number of marker effects to estimate should be proportional to the effective population size. Therefore, to maintain a constant ratio of the training population size to the number of effects estimated, the training and effective population sizes need to scale together (Meuwissen 2009). Notwithstanding this, managing a large population size is resource-intensive and sometimes may cause greater experimental errors. Strategies to tackle large populations include DNA marker-based solutions to type large populations or pooled bulks/selective extremes, high-throughput phenotyping facilities leading to the acquisition of accurate datasets in fields (Pandey et al. 2016; Xu et al. 2017).

#### Selection targets, methods, and priorities

Selection methods can be evaluated by measuring accuracy, a major component of the response to selection equation, *R* = *i* × *r* × *σ*_*g*_*/t*, where R is the response to selection, *i* is the selection intensity, *r* is the accuracy, and *σ*_*g*_ is the genetic standard deviation (Falconer and Mackay 1996). Since *r* is the square root of *h*^*2*^, the accuracy of selection in breeding can be improved by several means including the repeatability, greater number of trials, and high-throughput phenotyping of individuals.

A suite of basic traits or ‘unique selling propositions’ that the breeder needs to retain in future crop products can greatly inform selection priority in any breeding programs. The market will most likely reject any new variety/product without these basic (or must have) traits because the basic traits had contributed to wider acceptance of the established product, a popular existing variety in this case. Value-added/ game-changing traits can also be listed in selection priorities, which are not yet available to the elite market-oriented breeding program. These traits have enough potential for market or value-chain transformation to warrant additional investment in their development. Recent high-throughput phenotyping (HTP) platforms that facilitate accurate measurements on large populations for an array of morphological and physiological traits conferring climate resilience are noteworthy (Jin et al. 2020). Because of its scalability, HTP improves selection intensity in field-based phenotyping, whereas HTP facilitates early selection on single plant basis in controlled conditions. A marked reduction in the time invested in early selection cycle could contribute to improved genetic gain. The implementation of HTP will be more rewarding when used for harnessing correlated response through indirect selection (Y) or index selection (I) for the target trait (X) provided measurement of the target trait (X) using conventional means is inaccurate, costly and time-consuming (Morota et al. 2019). Recent examples that establish the superiority of HTP for indirect selection include selection of grain yield in wheat using near-infrared (NIR)-based spectral indices (Hu et al. 2020) and quantification of the intensity of *Septoria tritici* blotch disease in wheat based on canopy hyperspectral data (Yu et al. 2018). Improved selection intensity achieved by the HTP could thus accelerate genetic gain even when the heritability of the indirectly selected trait is not higher than the target trait.

### Enhancing trait heritability

Heritability investigates the relationship between observed/phenotypic values with phenotypic variance (*σ*^*2*^_*p*_) and their respective underlying true genotypic values (*g*) with genotypic variance *σ*^*2*^_*g*_. Further, *g* and *σ*^*2*^_*g*_ can be dissected into additive, dominance, and epistasis components to extract the average effects of alleles and breeding values (a), with variance *σ*^*2*^_*a*_. Depending on whether total genotypic variance (genotypic values) or additive genetic variance (breeding values) are considered, we refer to broad-sense heritability (*H*^*2*^) or narrow-sense heritability (*h*^*2*^), respectively (Schmidt et al. 2019).

#### Heritability increasing criteria

**Fig. 3:**
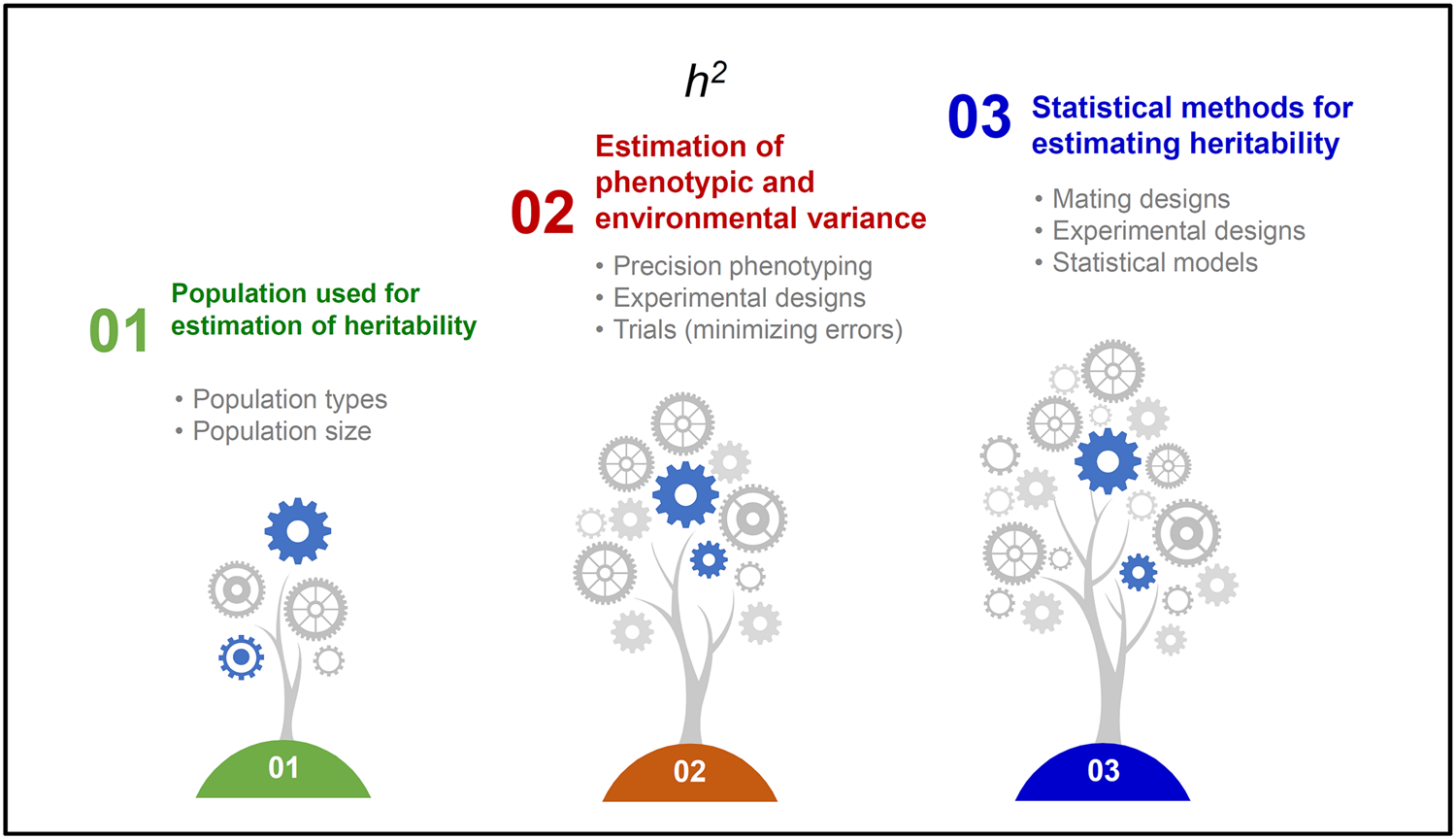
Enhancing trait heritability. Heritability of the target trait (*h*^*2*^) and its estimation can be improved via precision phenotyping and through minimizing experimental errors. Analysis of heritability mostly depends on population type (bi-parental and multi-parental, generation of population, and population size) and how we estimate the phenotypic and environmental variance. Precise phenotyping of trials/traits and reducing the experimental errors through the efficient statical design of trials. Proper statistical analysis of the datasets is a key to calculate the precise heritability of the trials and depends on use of models, parameters, and design used for the analysis

Additive genetic variance responds to selection. Also, non-additive components can be exploited but not in a recurrent manner. As reviewed in Xu et al. (2017), heritability estimates depend on a variety of factors including the size and the type of population used for estimation of phenotypic and environmental variances in addition to the statistical methods. Increasing the number of replications and locations has been reported to contribute towards an increase in heritability. Since the heritability does not respond linearly to an increase in replications, increasing the number of target locations for evaluations is considered a better option to increase heritability (Cobb et al. 2019). In this context, the adoption of partially replicated trials (p-rep) or the un-replicated designs may be beneficial in cases where spatial adjustments can be done properly (Fig. 3).

#### Minimize experimental errors

As mentioned in the earlier section, screening large populations across diverse agro-ecologies that portray the diversity of the TPEs faces a challenge of inflated experimental errors due to soil and spatial heterogeneity and strong G × E interactions. In wheat, prediction accuracies were improved with models that account for spatial adjustments (Lado et al. 2013). The conductance of large-scale multi-location trials (METs) in concert with environmental typing could enable the estimation of effects credited to the environment and G × E interactions (Fig. 3).

### Shortening breeding cycle time

**Fig. 4:**
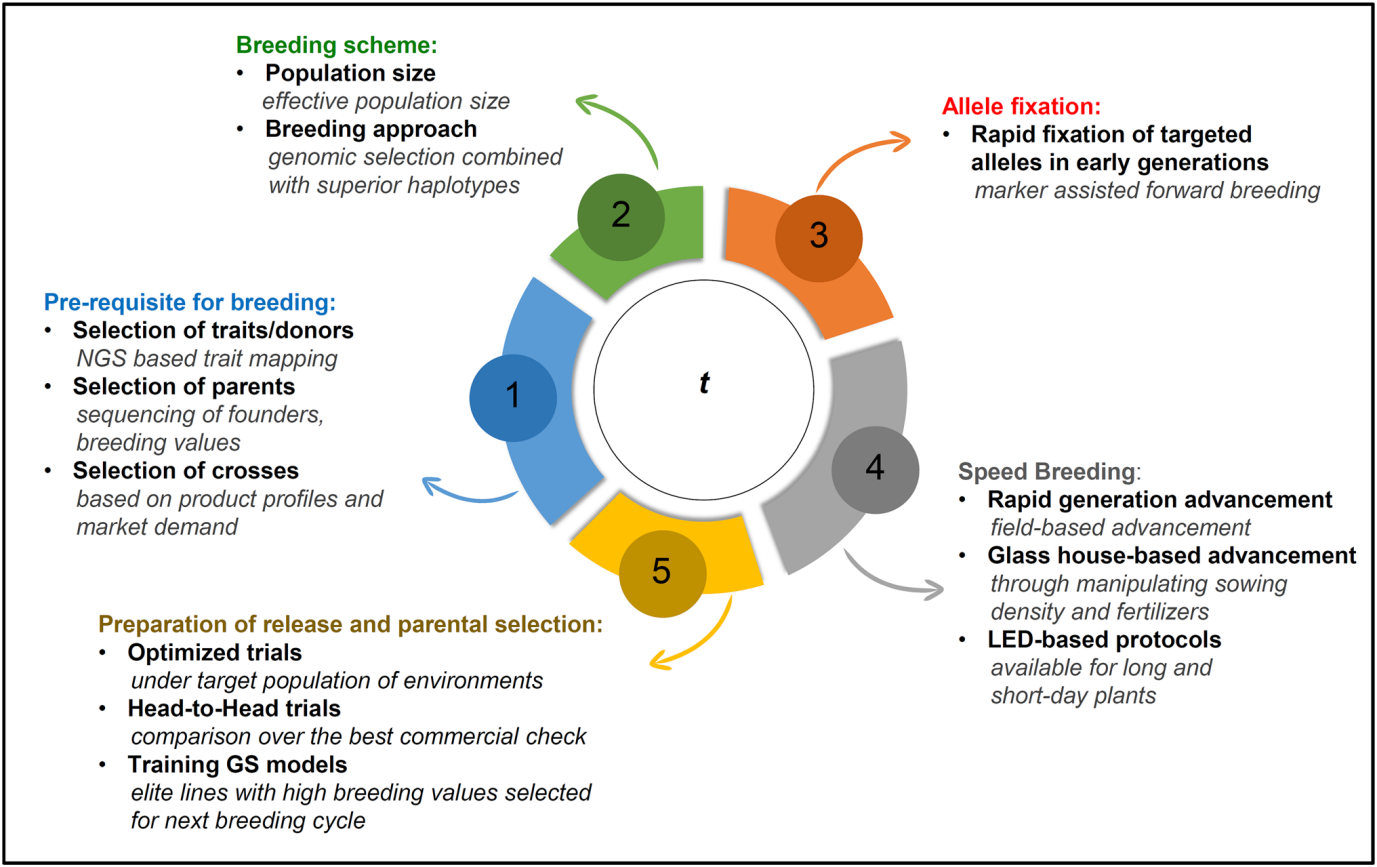
Accelerating the breeding cycle time (*t*). The breeding cycle starts with the selection of traits/donors/parents for the crossing programs based on the market demands which can be selected through precise phenotyping and WGRS for identification of appropriate genes/alleles/haplotypes. A genomic selection-based approach with appropriate population size can be adopted to develop lines with higher genetic gain. Lines can be fixed quickly through rapid fixation of alleles through forward breeding approach. Speed breeding/rapid generation advancement (RGA) approaches can be utilized for the advancing generations in fields or under controlled conditions. The preparation of lines for commercial release is an important step in which phenotypic evaluation of lines under target population of environments (TPEs) is a crucial step together with head-to-head trials of newly developed lines with local and national checks. Data generated through these trials can be utilized in selection of elite parental lines and can inform genomic selection cycle. Genetic gain per unit time can be improved through incorporating the above-discussed points which shall be useful in shortened cycle time (*t*) by integrated breeding strategies

Accelerating the breeding process shortens the cycle time and thus increases the total genetic gain per year (Fig. 4). Reducing generation interval time has a greater impact on the rate of genetic gain as compared to the other components of a breeder’s equation including selection intensity, selection accuracy, and trait heritability (Araus et al. 2018). There are several ways to shorten the cycle time (Fig. 4). Firstly, the selection of appropriate parental lines for elite × elite crosses. Secondly, speeding up the process to reach homozygosity, which can be achieved by field-based rapid generation advance (RGA)/speed breeding (SB)/double haploid (DH) in fewer years/generations. By using above approaches, selected homozygous lines fixed for major alleles can be utilized for GEBV prediction in GS models. Integration of SB with GS has been referred to as Speed-GS (Voss-Fels et al. 2019)

#### Selection of parents and rapid breeding cycle

Generation of product profiles based on the market preference including inputs from all the stakeholders (consumer, millers, farmers, etc.) sets a prerequisite priority to commence any breeding programs. This can help design the prototype required for the market and breeder-preferred traits while developing new varieties. This step will ensure not only focus breeding activities on developing varieties/lines for specific regions but also rapid adoption after the release for commercial cultivation in any crop. The selection of the appropriate parents to be used in artificial crosses is one of the key breeding decisions that will facilitate the exploitation of maximum genetic variability and recovery of superior recombinants. Several techniques have been used to identify genotypes with promising and desirable agronomic traits for hybridization.

Speed-GS can accelerate the development of new lines based on product profiles by establishing haplotype-based breeding panels. Based on the region-based needs, which include donors for targeted traits (nutritional, biotic and abiotic stresses, etc.), high-yielding varieties for different ecologies and elite lines possessing superior haplotypes for the traits of interest could enhance genetic gains. These assembled panels can be grown in the TPEs for a minimum of two years for generating high-quality phenotypic data for the targeted traits and need to be genotyped at high-density. Based on the haplotypic and breeding values (gBLUP) and the targeted market-oriented demands, parental lines shall be selected for the generation of F_1_s. The true F_1_ plants confirmed by haplotype-specific SNPs are self-pollinated to generate large numbers of F_2_ seeds. Single seed descent (SSD) methods coupled with accelerated generation turnover under field or controlled conditions could lead to rapid fixing of the advanced lines for homozygosity. Selected fixed lines exhibiting phenotypic superiority can be further tested with trait-specific SNP panels for the selection of lines carrying maximum alleles of interest. The entire process from crossing to the development of the final set of lines for genotyping will demand a short period of < 2 years.

Lines with superior alleles selected using genotypic and phenotypic scores can be further subjected to GS analysis. Homozygous lines can be assayed on fixed arrays (SNP arrays) or low-cost sequencing-based genotyping platforms such as rAMPSeq (Buckler et al. 2016). Unlike cost-effective genotyping methods requiring greater analytical strength, genotyping of lines with an SNP array, though a bit costly, generates data that is less computationally demanding (Rasheed et al. 2017). However, from breeder′s perspectives the genotyping cost still needs a significant reduction to make it affordable to accommodate large numbers of lines for GS analysis (Longin et al. 2015). Instead of repeated phenotyping of advanced lines, GEBVs can be estimated through appropriate models. These lines with higher GEBVs can be utilized in two ways: (a) region-specific high GEBVs lines can be utilized as one the parent of GS-based breeding funnel to recombine more haplotypes/superior minor alleles to enhance the genetic gain in every recombination cycles, and (b) superior lines with high GEBVs can be tested in advanced yield trials in TPEs utilizing most advanced and cost-effective experimental design. High-performing lines identified in advanced yield trials can be further tested in multi-location environments for the identification of superior lines, which shall be tested in a coordinated research program of the national system.

#### Product release and delivering genetic gain in farmers’ fields

Promising lines can be further tested at a large scale (multi-location testing) including targeted phenotypic environments at farmer fields with their best performing checks. The best performing lines evaluated and identified in such ways will ensure high genetic gains (Varshney et al. 2018). Prolonged cultivation of old varieties on farmer’s fields has also been found to deteriorate the rate of genetic gain (see Bohra et al. 2020a). Equally importantly, the research community working on the extension aspect should vigorously demonstrate the advantages of the recently released product offers over the existing (or to be replaced) variety (Atlin et al. 2017).

## Summary and outlook

Enhancing genetic gain amid climate change necessitates breeding innovations to deliver the productivity gain not only in the research plots but in farmers fields’ as well. An integrated approach is required to realize the genetic gain through the modernization of the breeding programs. As discussed above, creation and use of favorable effect alleles into the breeding programs is required to enhance the genetic variance (*σ*^*2*^_*g*_). Elite/favorable alleles can be introduced into the breeding pipeline through the development of new parental lines with the allele of interest. The development of new breeding lines required optimized breeding programs that maintain a balance between the size of the population and the selection intensity (*i*). Better estimation of trait heritability (*h*^*2*^) remains crucial to improve the rate of genetic gain. In this context, advances in the statistical methods and experimental designs could contribute to improving heritability estimates. Likewise, accelerating the breeding cycle time (*t*) significantly influences selection response. Thus, informed choice of appropriate parents, optimized breeding pipelines, rapid fixation of target alleles, and combining SB/RGA into breeding programs present potential ways to enhance breeding efficiency. Deployment of these approaches in the modernized breeding programs will help realizing the higher genetic gains in fields for the small-holder farmers in the developing world.
